# Relevance of secretor status genotype and microbiota composition in susceptibility to rotavirus and norovirus infections in humans

**DOI:** 10.1038/srep45559

**Published:** 2017-03-30

**Authors:** Jesús Rodríguez-Díaz, Izaskun García-Mantrana, Susana Vila-Vicent, Roberto Gozalbo-Rovira, Javier Buesa, Vicente Monedero, Maria Carmen Collado

**Affiliations:** 1 Department of Microbiology, School of Medicine, University of Valencia, Valencia, Spain; 2 Department of Biotechnology, Institute of Agrochemistry and Food Technology, National Research Council (IATA-CSIC), Valencia, Spain

## Abstract

Host genetic factors, such as histo-blood group antigens (HBGAs), are associated with susceptibility to norovirus (NoV) and rotavirus (RV) infections. Recent advances point to the gut microbiome as a key player necessary for a viral pathogen to cause infection. *In vitro* NoV attachment to host cells and resulting infections have been linked to interactions with certain bacterial types in the gut microbiota. We investigated the relationship between host genotype, gut microbiota, and viral infections. Saliva and fecal samples from 35 adult volunteers were analysed for secretor status genotype, the gut microbiota composition by 16S rRNA gene sequencing, and salivary IgA titers to NoV and RV. Higher levels of IgA against NoV and RV were related to secretor-positive status. No significant differences were found between the FUT2 genotype groups, although the multivariate analysis showed a significant impact of host genotype on specific viral susceptibilities in the microbiome composition. A specific link was found between the abundance of certain bacterial groups, such as *Faecalibacterium* and *Ruminococcus* spp., and lower IgA titers against NoV and RV. As a conclusion, we can state that there is a link between host genetics, gut microbiota, and susceptibility to viral infections in humans.

Acute gastroenteritis (AGE) is a major worldwide health issue, associated with a high economic burden in developed countries and high annual mortality, particularly affecting children, in developing countries^1^. The major etiological agents of viral AGE in children are rotaviruses (RVs) (90% represented by RV group A [RVA])^2^ and noroviruses (NoVs)^1^.

It was previously shown that susceptibility to NoV infections differed between individuals^3^ and was associated with histo-blood group antigens (HBGAs)^4^. It has been suggested that HBGAs expressed on epithelial surfaces function as receptors for NoV, with different NoV strains showing different properties with regard to the ability to bind to different HBGAs^5^. Non-secretor individuals (lacking both functional FUT2 alleles) do not express H-antigen structures (Fucα1,2-Galβ1,3-GlcNAcβ1,3-Gal) on their mucosa and are less susceptible to NoV^3^. Based on the findings of recent publications^6^,^7^, the determination of susceptibility to RV infections should also consider HBGA phenotypes. Several studies have suggested that the non-secretor phenotype was restrictive to P[8] and P[4] RV genotype infections, as revealed in analyses of symptomatic infections^6^,^8^,^9^,^10^,^11^ or specific seral IgG levels^12^.

The gastrointestinal environment is a very complex ecosystem that contains a vast bacterial population in terms of numbers and diversity^13^. This population varies between individuals and is subject to changes depending on diverse factors, such as genetics, diet, and health status. Intestinal bacteria have a vast enzymatic potential for scavenging diet and host glycans (e.g., carbohydrate structures from *O*-glycosylated mucins and other HBGA structures on mucosal surfaces)^14^. Furthermore, the high L-fucose concentration in the gastrointestinal tract plays an important role in the ecology of this niche, assisting in commensal colonization^15^,^16^. Intestinal microbiome analyses have also revealed differences in the microbiota structure based on FUT2 status, with non-secretors showing reduced microbial richness compared to secretors^17^,^18^.

Some intestinal viruses rely on the microbiota for infectivity. No cell-culture system had previously allowed the *in vitro* replication of human NoV until recent findings showed that the NoV GII.4 genotype can infect human lymphocytic B cell line if an accompanying intestinal microbiota is present^19^. Moreover, NoV replication has been recently achieved in human enteroids derived from stem cells^20^. There are also evidences for microbiota-dependent RV infections in a study in which the use of germ-free animals or antibiotic treatments resulted in a >40% reduction of viral infections in a mouse model^21^.

In the present study, we analysed the link between secretor status, the gut microbiota, and susceptibility to RV and NoV infections in healthy individuals, measured via salivary IgA titers to RV and NoV. The expected link between secretor status and susceptibility to RV and NoV was found, and multivariate analyses showed that secretor status, the microbiota, and viral susceptibility are interdependent for both RV and NoV. Finally, specific correlations were found between certain bacterial groups and the risk of RV and NoV infections.

## Results

### Secretor status correlated with salivary anti-NoV and anti-RV titers

**Figure 1:**
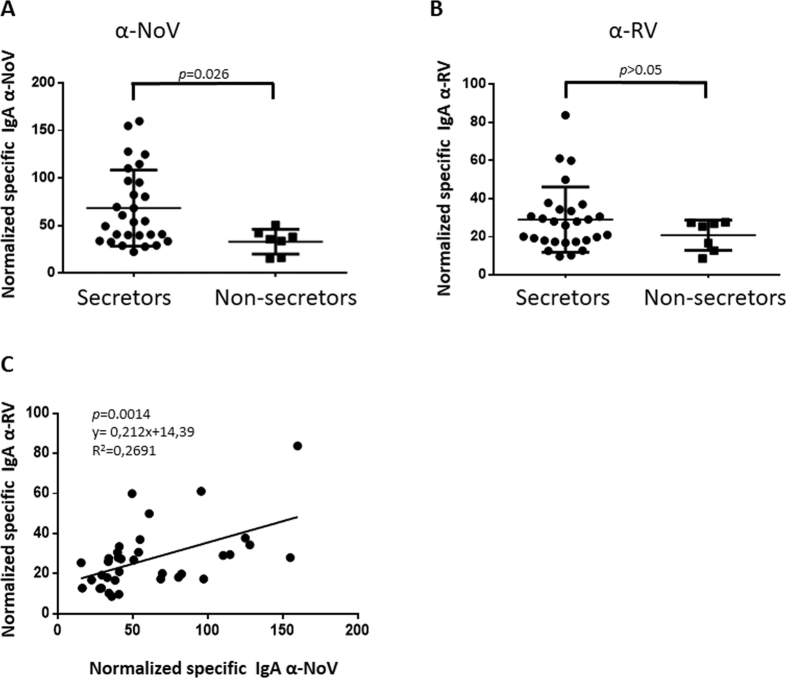
Normalised specific salivary IgA against norovirus (NoV) (panel A) and against rotavirus (RV) (panel B) in a population of 28 secretor (9 FUT2^+/+^ and 19 FUT2^+/−^) and 7 non-secretor (FUT2^−/−^) individuals. The correlation between the IgA titers in both viruses is shown in panel C.

The aim of the present study was to identify relationships between viral susceptibility, host genetic factors, and the intestinal microbiota. To avoid invasive procedures, salivary IgA titers to NoV and RV were obtained. The proportion of non-secretor individuals in the studied population was 22.8%, which is in accordance with the prevalence of this phenotype in Caucasian populations (20%). The observed frequencies of the three genotypes (FUT2^+/+^(27.2%), FUT2^+/−^ (50%) and FUT2^−/−^ (22.8%) are in concordance with the Hardy-Weinberg equilibrium (*p* = 0.5974 for the deviation from the null hypothesis). The results confirmed that normalised salivary IgA titers against NoV are significantly higher (*p* < 0.05) in secretor-positive (FUT2^+/+^ or FUT2^+/−^) individuals than in non-secretor individuals (FUT2^−/−^) (Fig. 1A), as has been previously described based on analyses of serum IgG titers^22^. When normalised salivary anti-RV IgA titers were analysed, no significant differences were found between the groups, but the same tendency with regard to NoV was observed (Fig. 1B). The antibody titers against RV and NoV presented a positive correlation (*p* < 0.01) indicating that there is a relationship in the susceptibility to both viruses (Fig. 1C)

### Microbiome composition

**Figure 2:**
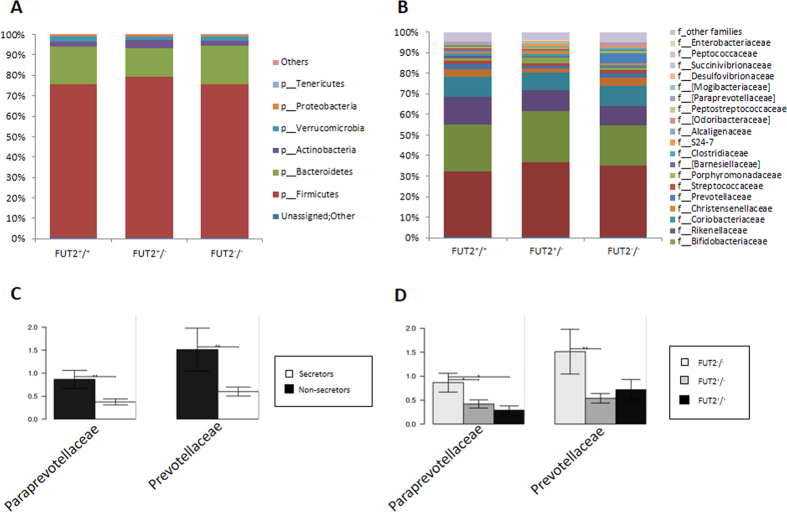
Relative abundance in % of the top phyla (Panel A) and families (Panel B) in the studied population including 7 non-secretor individuals (FUT2^−/−^), 9 homozygous secretor (FUT2^+/+^) and 19 heterozygous (FUT2^+/−^) individuals. Significantly different taxa at family level between secretor and non-secretors (Panel C) and FUT2 genotypes (Panel D) are shown as bar chart (p < 0.05, ANOVA). Standard error is depicted by error bars. Pair-wise comparisons are done by t-test and annotated as *p < 0.05, **p < 0.01.

A total of 2,456,381 quality-filtered 16S rDNA sequences were obtained from the subjects’ feces, with an average of 70,182.3 ± 37,784.2 sequences per sample. These readings were clustered into 52,769 unique operational taxonomic units (OTUs), with an average of 1,500 OTUs per subject. The most abundant bacterial phyla were Firmicutes (76.01%), Bacteroidetes (17.27%), Actinobacteria (2.85%), and Verrucomicrobia (1.98%). Proteobacteria were present in less than 1%. At family level, the most predominant bacteria belonged to the *Ruminococcaceae* (35.58%), *Lachnospiraceae* (23.02%), *Bacteroidaceae* (11.36%), and other Clostridiales (9.66%), followed by *Veillonellaceae* (3.43%) and *Verrucomicrobiaceae* (2.00%) (Fig. 2).

No significant differences were found between the different FUT2 allele groups (FUT2^+/+^, FUT2^+/−^ and FUT2^−/−^) at the phylum level (Fig. 2A). However, significantly higher levels were found in non-secretors (FUT2^−/−^) for the families *Prevotellaceae* and *Paraprevotellaceae* compared to secretors (FUT2^+/+^, FUT2^+/−^) (Fig. 2B,C and D). At the OTU level, we found significant differences in 16 OTUs, mostly belonging to the Firmicutes and Bacteroidetes phyla; these were predominantly more abundant in the non-secretors except for OTU *Bacteroides_51792*, which was more abundant in the secretors (Supplementary Figure S1). Six OTUs were different between the genotype groups (Supplementary Figure S2). UNIFRAC plots did not show different clusters between secretor and non-secretor (not shown). The Adonis analysis (permutational MANOVA [PERMANOVA]) showed no significant differences at the OTU level between the FUT2 groups (*p* = 0.179).

An alpha diversity analysis was performed on the samples after rarefaction to 12,100 sequences/sample (minimum sampling depth). Rarefaction curves generated for the Chao1 and Shannon indexes showed no differences between genotype groups (Supplementary Figure S3). Predictive functional profiling (PICRUSt) showed no significant differences between FUT2 groups and genotypes (not shown). The most predominant bacterial genes were related to metabolism, genetic information processing, and environmental information processing. We also performed LEfSe tests to detect KEGG pathways, with significantly different abundance levels in the samples, but we did not find significant differences between the secretor and non-secretor samples (LDA > 2, *p* > 0.05).

### Intestinal microbiota correlates with susceptibility to RV and NoV

**Figure 3:**
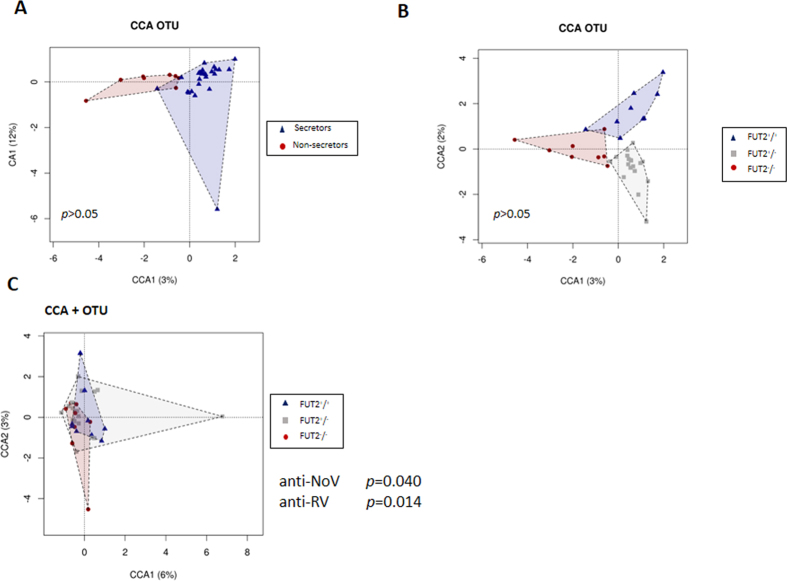
Multivariate analysis plots based microbiota composition at Operational Taxonomic Units (OTU) level between secretors (blue) and non-secretors (red) (Panel A) and also, between FUT2 genotypes (FUT2^+/+^ = blue, FUT2^+/−^ = grey, FUT2^−/−^ = red) (Panel B). Canonical correspondence analysis (CCA+) showing the relationship between gut microbiome composition of the FUT2 genotypes and both anti-virus IgA titers, anti-RV and anti-NoV respectively (Panel C). Anti-virus IgA titers significantly explain variations observed in the gut microbiota (*p* < 0.05).

A multivariate canonical correspondence analysis (CCA) on the observed OTUs was performed. The plot showed clearly different groups in the microbial compositions between the non-secretors and the secretors at the phenotype and the genotype level although without significant differences (Fig. 3A and B, *p* > 0.05).

We found that both, anti-NoV and anti-RV IgA levels, significantly explained the variations in the gut microbial composition associated to FUT2 secretor status (*p* = 0.040 and *p* = 0.014, respectively) on the CCA + analysis (Fig. 3C), confirming the hypothesis that all three factors (secretor status, microbiota, and susceptibility to RV and NoV) are interdependent.

**Figure 4:**
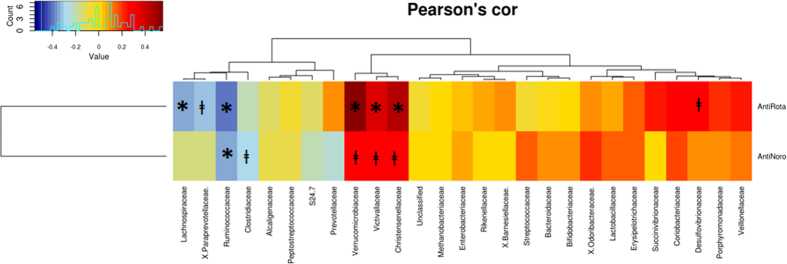
Pearson correlations heatmap between the susceptibility to viral infection to rotavirus (RV) and norovirus (NoV) (measured as anti viral IgA titers) and the relative abundances of specific bacteria at family level. R and *p*-values are indicated in the table. Sequences > 1% were included in the analysis. *p < 0.05 and ^ⱡ^*p* < 0.09.

**Figure 5:**
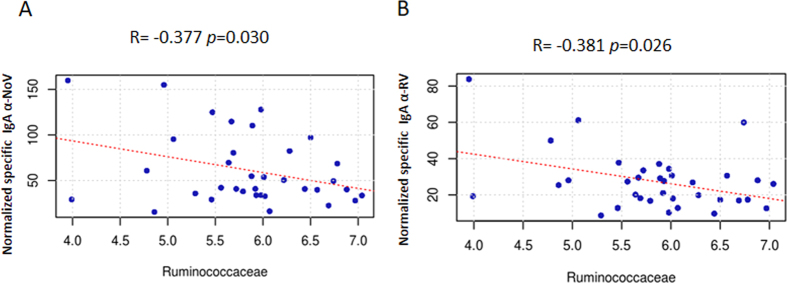
Pearson correlation plots at family level and the susceptibility to norovirus (NoV) (Panel A) and rotavirus (RV) (Panel B). The results show significantly associated correlation between the *Ruminococcaceae* family with Anti-IgA levels (p < 0.05). A scatter plot is shown plotting the abundance of the *Ruminococcaceae* family (x-axis) versus the anti-IgA titers (y-axis).

**Figure 6:**
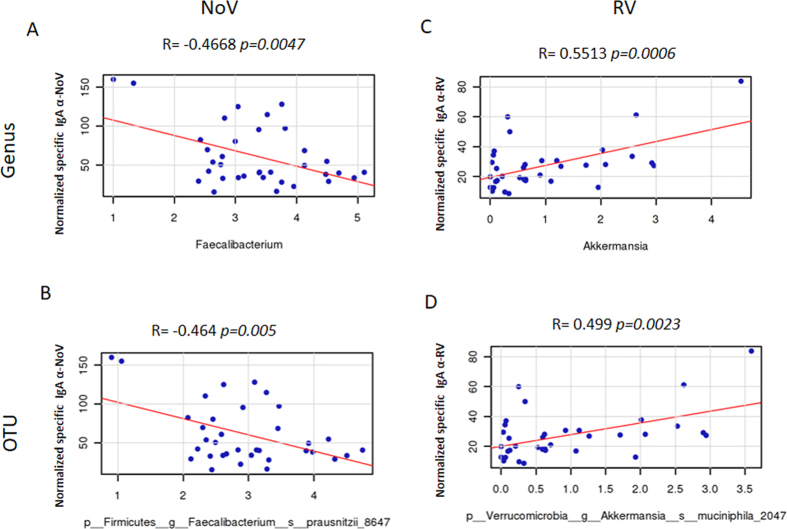
Pearson correlation plots at genus and OTUS levels and the susceptibility to norovirus (NoV) (Panels A and B) and to rotavirus (RV) (Panels C and D). The *p*-values computed by correlation are reported indicating if the specific genus or OTU is significantly associated with Anti-IgA levels (*p*-values are indicated in each graphic).

In order to seek out the relationships between specific bacterial groups and susceptibility to RV and NoV infections in humans, a Pearson correlation analysis was performed showing a potential link between gut microbiota composition and the susceptibility to RV and NoV infection (Fig. 4). The results showed a significant negative correlation between susceptibility to NoV (R = −0.377, *p* = 0.03) and RV infection (R = −0.381, *p* = 0.026) infection, with the number of bacteria from the *Ruminococcaceae* family (Fig. 5A and B, respectively) indicating that individuals with a greater abundance of *Ruminococcaceae* bacteria might have lower susceptibility to both types of viral infection. At genus level, a negative correlation between susceptibility to NoV and the abundance of *Faecalibacterium* (R = −0.4668, *p* = 0.0047) was observed (Fig. 6A). This correlation was confirmed at the OTU level, where a negative correlation (R = −0.464, *p* = 0.005) was found for NoV susceptibility and for the OTU *F. prausnitzii_8647* (Fig. 6B). Another significant correlation was found between the OTU *Ruminococcaceae_23936* (R = −0.3811, *p* = 0.024) and susceptibility to NoV. Different results were found among the different bacterial groups with regard to susceptibility to RV; a positive correlation was found at the genus level (R = 0.5513, *p* = 0.0006) between *Akkermansia* and susceptibility to RV (Fig. 6C). This correlation was confirmed at the OTU level, where a positive correlation was found between *Akkermansia muciniphila_2047* and susceptibility to RV (R = 0.499, *p* = 0.0023) (Fig. 6D), indicating that the abundance of this bacterium correlates with increased susceptibility to RV infections. Other bacterial groups also correlated negatively with susceptibility to RV at the genus level, including *Paraprevotella* (R = −0.368, *p* = 0.03) and *Faecalibacterium* (R = −0.3319, *p* = 0.051). However, this last correlation did not reach significance. A similar level of significance was also seen at the OTU level, where *F. prausnitzii_38832* showed a negative correlation with susceptibility to RV infection (R = −0.321, *p* = 0.06). A negative correlation was found between two different *Ruminococcaceae* OTUs, *Ruminococcaceae_44932* (R = −0.3623, *p* = 0.032) and *Ruminococcaceae_20617* (R = −0.357, *p* = 0.035), and susceptibility to RV infections.

## Discussion

Recent research findings on the involvement of intestinal bacteria in NoV and RV infections have established that the microbiota plays a pivotal role in viral susceptibility^19^,^21^. Interestingly, this association exists even if these viruses replicate in the small intestine, whereas most of the intestinal bacteria are present in the colon. Susceptibility to NoV and RV infections has been related to HBGA types, but individual secretor status, linked to the FUT2 gene, is of special relevance. The presence of anti-RV and anti-NoV antibodies can be used as indicator of previous infections, constituting an indirect marker of virus susceptibility. We have seen that levels of specific secretory immunoglobulins (such as IgA) in the saliva relate to the secretor phenotype for NoV. This confirms the relevance of FUT2 in viral infectivity and indicates that, similar to humoral responses (serum IgG^22^), the secretory antibody response also serves as a marker for susceptibility. A trend-like correlation was only observed for RV, which may account for the higher variability of RV depending on secretor status, which is based on viral genotype, as has been described for P[8] and P[6] RV variants^9^.

NoV and RV infections depend on the gut microbiota^19^,^21^ and on secretor status, while the gut microbiota is also simultaneously dependent on secretor status. Previous studies reported that the non-secretor phenotype was associated with a lower intestinal microbial diversity^17^,^23^,^24^. A study also showed differences in microbiota between homozygote secretors (FUT2^+/+^) and heterozygote secretors (FUT2^+/−^)^18^. Therefore, a dose effect of FUT2, as has already been described for NoV attachment^25^, cannot be excluded. In our study, differences in the microbiota could be established, although in contrast to these previous studies, no difference in microbial richness between secretor and non-secretor individuals was evidenced. A trend-like difference was only reported at the genus level for changes in microbial communities between FUT2 genotypes in a work by Wacklin *et al*.^17^. Similarly, our multivariate analysis at the OTU level did not show statistically significant differences between FUT2 genotypes although group clusters were clearly identified. A recently published study involving a large population also failed in finding an association of intestinal microbial composition with blood group (ABO) or secretor status^26^. In that study, however, other factors that could determine how FUT2 status interacts with the intestinal microbiota have to be considered, as the studied group was composed of female tweens with and average age of 61. Therefore, differences in physiology, diet, or activity level between different age groups may lead to microbiome composition differences that could mask an association with FUT2^26^. Diet has been identified as a major factor influencing intestinal microbial composition^27^. Interactions of dietary factors that depend on the geographic origin of the studied individuals (Spain in our case and Finland, Germany, USA and UK in the previous studies^17^,^18^,^26^,^28^ with secretor status may explain the distinct contribution of FUT2 to the composition of the microbiota, as found in our study. In this sense, it has been shown that differences in intestinal microbial composition between wild-type and FUT2^−/−^ mice with humanised microbiota are eliminated when the animals follow a diet deficient in polysaccharides^24^. Similar to this diet by phenotype (FUT2) effect on the microbiota, disease by secretor status effects on microbial composition have been reported for intestinal inflammatory diseases (i.e. Crohn disease^28^), when comparing healthy and diseased individuals. This suggests that FUT2 status may play a role in shaping the intestinal microbiome in certain diet and disease contexts.

Although the number of analysed subjects in our study (35 individuals) and the previous works (39^18^, 24^17^, and 18^28^ individuals and mice with humanised microbiota from a single donor^24^), with the exception of the mentioned female tweens study^26^, are low, FUT2 still emerges as a genetic factor modulating the intestinal microbiota. Notwithstanding, in addition to the limitation of the low number of samples, our study may present other weaknesses. Possible bias introduced by the use of antibiotics in the period prior to the last two months before sampling (our selection criteria) or by the smoking or food habits of the participants, cannot be excluded, since all they can impact the microbiota composition. The gender of volunteers (63% women, 37% men) may also act as a confounder, since male and female can display differences in microbiota composition^29^. However, we showed that there were no differences in microbiota richness and composition based on gender (Supplementary Figure S4). In addition, RV causes diarrhoea mostly in the young and in the present study only adults (average age of 38) were enrolled. Adults can also be infected by RV without symptoms, but studies with larger populations of infants, especially prospective studies with large cohorts are needed to obtain more robust results. Specifically, it will be necessary to establish the host-microbiota-viral susceptibility/protection relationship in the RV disease susceptible population (<5 years).

In spite of the exposed limitations, the inclusion of anti-NoV and anti-RV IgA levels in our analyses significantly explained the inter-individual variations in the gut microbial composition associated with FUT2 status (Fig. 3C). This susceptibility by phenotype effect on the microbiota reinforces the idea that the three factors are interconnected. Also, remarkable similarities were found between our study and previous works. In agreement with former reports, we identified specific microbiota associations according to secretor status. In the present study, members of *Prevotellaceae* were detected in greater numbers in non-secretors, and *Prevotella* OTUs and the *Prevotella* group have been previously associated with the non-secretor phenotype by 16S rDNA sequencing^28^ and FISH^23^, respectively. Similar to our results, members of the genus *Bacteroides* and the family *Lachnospiraceae* were more abundant in non-secretor mice with humanised microbiota^24^, and increased numbers of Bacteroidetes have also been linked to non-secretors in two human studies^18^,^28^. However, two *Bacteroides* species (*B. plebeius* and *B. fragilis*) were related to the secretor phenotype on a PCR-DGGE analysis^17^. These species are characterised as having relatively high numbers of enzymes (i.e. α-L-fucosidases and other glycosyl hydrolases^30^), so they can take nutritional advantage of the degradation of HBGA glycan structures. Two different factors may be involved in the modulation of the microbiota by the availability of fucosylated carbohydrates: (i) L-fucose can be used as a carbon source in some intestinal bacteria, promoting their growth, and (ii) fucosylated HBGAs can act as specific bacterial binding sites that promote attachment and persistence in the gut. These two factors may have a complex relationship, as carbohydrate cross-feeding is common in the intestine, where some bacteria may benefit from the degradation of HBGA-glycan structures by other members of the microbiota^31^. Furthermore, it has been established that the intestinal microbiota itself affects host intestinal glycosylation patterns^32^,^33^, including fucosylation.

Of note, we were able to establish correlations between different taxa and susceptibility to both NoV and RV (i.e., *Ruminococcaceae*). NoV and RV infectivity was negatively correlated with different OTUs of *Faecalibacterium* and *Ruminococcaceae*. Conversely, *Akkermansia* levels correlated to higher levels of anti-RV IgAs. The only species of this genus, *A. muciniphila*, possesses a remarkable capacity for feeding on host mucins^34^, and trends toward a lower relative abundance in non-secretors^17^. Our data showed that *Akkermansia* was not different between the FUT2 groups, but was more abundantly present in non-secretors compared to secretors (1.08% vs 0.95%, respectively). In this context, the intriguing lack of efficacy of RV oral vaccines (consisting of live-attenuated viruses) in low- versus high-income countries may be based on differences between the settings that impact the intestinal microbiota^35^. However, the role of different bacterial taxa in RV/NoV infectivity still remains elusive, and establishing causal effects is still not possible. In investigations on human NoV infections of B cells, Jones *et al*. showed that *Enterobacter cloacae* expressed H-type substances on their surface that promoted viral binding to target cells and allowed *in vitro* infectivity^19^, while Miura *et al*. identified HBGA-like substances from *Enterobacter spp.* that promote cellular attachment of NoV viral-like particles^36^. In contrast, recent results with a gnotobiotic pig model questioned the role of this bacterium, demonstrating that *E. cloacae*-colonised pigs antagonised human NoV (GII.4) infections, resulting in reduced viral shedding^37^.

Understanding the interactions between virus, host, and microbiota could open new avenues in personalised medicine. Many possibilities exist in this context, including the rational design of NoV or RV vaccine compositions/protocols based on specific interactions between viral strains/bacteria and the host genotype, or the implementation of dietary strategies (e.g., pre- or probiotics) that target specific bacterial groups, resulting in enhanced defences. In this regard, recent results have shown that the anti-NoV effect of vitamin A supplementation can be partly explained through microbiota modulation, which results in an increased number of *Lactobacillus*, leading to immunomodulatory effects mediated by IFN-β^38^. Altogether, the findings show that viral infectivity must be understood within a holistic framework, in which host genetic background, glycobiology, and gut microbiota cannot be separated from viruses and host cells.

## Methods

### Subjects and Sampling

A total of 35 Spanish volunteers with a mean age of 38.43 ± 9.14 years and normal weight (body mass index [BMI] = 23.59 ± 3.68 kg/m^2^) participated in this study (Table 1). No gastrointestinal disorders, use of medicines (including probiotics and antibiotics) in the last two months, or any other diseases or problems were reported by the participants, who were given oral and written instructions for the standardised collection of samples. Stool samples were collected and kept frozen at −20 °C until delivery to the laboratory. Saliva samples were collected in the morning (8–10 a.m.) to minimize the effects of the circadian rhythm. The participants were instructed not to smoke, eat, drink, or brush their teeth for 2 h before the saliva collection.

### Ethic statement

Written informed consent was obtained from all participants, and the study protocol was approved by the local ethics committee of the Atencion Primaria-Generalitat Valenciana (CEIC-APCV). We confirm that all methods were performed in accordance with the relevant guidelines and regulations.

### FUT2 genotyping

Host DNA was extracted from saliva samples using the JETQUICK Blood & cell culture DNA spin kit (Genomed) following the manufacturer instructions. The secretor status was investigated by genotyping the FUT2 gene by PCR-RFLP as described previously^25^.

### Determination of anti-RV and anti-NoV titers in saliva samples

To determine salivary IgA titers to RV and NoV, microtiter plates were coated either with purified RV triple layered particles (TLPs)^39^ (2 μg/ml) from human Wa strain (G1P[8]) or with a mix of norovirus P particles from genotypes GI.1, GII.4 (VA386_1996 and Den Haag_2006b variants) and GII.9 produced as previously described^40^,^41^ at 1 μg/ml each, in carbonate/bicarbonate buffer (pH 9.6) and incubated at 4 °C overnight. Negative controls wells were coated with BSA at 1 μg/ml in carbonate/bicarbonate buffer. Plates were washed three times with PBS containing 0.1% Tween 20 (PBS-T) and blocked for 1 h at 37 °C with PBS-T with 3% BSA. After blocking, plates were incubated in triplicate with saliva samples at a 1:100 dilution in PBS-T with 1% BSA for 1.5 h at 37 °C and NoV- and RV-specific IgAs were detected with a secondary anti-human IgA antibody conjugated with HRP (Sigma) at a 1:4,000 dilution in PBS-T with 1% BSA for 1 h at 37 °C. Wells were washed four times with PBS-T, and the bound antibody was detected by the addition of 50 μl of *o*-phenylenediamine (Sigma). The reaction was stopped at 10 min with 3 M H_2_SO_4_, and the absorbance was read at 492 nm (Multiskan FC spectrophotometer, Thermo Scientific). Microtiter plates were simultaneously coated with a 1:1,000 dilution of saliva in carbonate/bicarbonate buffer and processed in the same way as above to obtain the total IgA content of each saliva sample. The OD_492_ of the negative controls (BSA) were subtracted to obtain the specific anti-RV or anti-NoV titer. The OD_492_ of the specific anti-RV or anti-NoV was divided by the OD_492_ of total IgA and multiplied by 100 to obtain the normalised specific salivary IgA titer for each virus. All measurements were taken within the lineal range of the instrument (OD_492_ from 0.05 to 1.5).

### Fecal DNA extraction and 16S rDNA sequencing

Total DNA was isolated from the fecal pellets by using the MasterPure Complete DNA & RNA Purification Kit (Epicentre) according to the manufacturer’s instructions with some modifications, including a bead-beater step and enzyme incubation to increase DNA extraction. The specific DNA extraction protocol was described previously^42^. DNA concentration in samples was measured using a Qubit^®^ 2.0 Fluorometer (Life Technology, Carlsbad, CA, USA) and diluted to 5 ng/μL. The V3-V4 region of 16S rDNA gene was amplified by PCR using Illumina adapter overhang nucleotide sequences following Illumina protocols. After 16S rDNA gene amplification, the mutiplexing step was performed using Nextera XT Index Kit. 1 μl of the PCR product was checked with a Bioanalyzer DNA 1000 chip and libraries were sequenced using a 2 × 300pb paired-end run (MiSeq Reagent kit v3) on a MiSeq-Illumina platform (FISABIO sequencing service, Valencia, Spain). To rule out and control for possible reagent contamination, reagents for DNA extraction and for PCR amplification were also sequenced as controls.

### Bioinformatics and Statistical analysis

Quality assessment was performed by the use of prinseq-lite program^43^ applying following parameters: min_length: 50; trim_qual_right: 20; trim_qual_type: mean; trim_qual_window: 20. R1 and R2 from Illumina sequencing where joined using fastq-join from ea-tools suite^44^. Data were obtained using an ad-hoc pipeline written in RStatistics environment^45^ and data processing was performed using a QIIME pipeline (version 1.9.0)^46^. Chimeric sequences and sequences that could not be aligned were also removed from the data set. The clustered sequences were utilised to construct Operational Taxonomic Units (OTUs) tables with 97% identity and representative sequences were classified into the respective taxonomical level from phylum to genus based on the Greengenes 16S rRNA gene database. Sequences that could not be classified to domain level, or were classified as Cyanobacteria, were removed from the dataset as they likely represent ingested plant material. All communities were rarefied to 12,100 reads per sample to calculate bacterial diversity. Subsequently, alpha diversity indexes (Chao1 and Shannon) and beta diversity using UniFrac distance among samples and PERMANOVA^47^ was used to test significance. Calypso software version 5.2 (http://cgenome.net/calypso/) was used with data transformed by square root with total sum normalization for the statistical analysis, multivariate test and data mining. To assess the relationship between FUT2 genotypes and the microbiota at family, genus and OTU levels the ANOVA test was utilized. Pearson’s correlations between the levels of bacterial families and antibody levels were also calculated and plotted using Calypso software. Predictive functional analysis was performed using PICRUSt^48^ with Kyoto Encyclopedia of Genes and Genomes (KEGG) and linear discriminant analysis effect size (LEfSe)^49^ was used to detect unique biomarkers (LDA score > 3.0).

## Additional Information

**How to cite this article:** Rodríguez-Díaz, J. *et al*. Relevance of secretor status genotype and microbiota composition in susceptibility to rotavirus and norovirus infections in humans. *Sci. Rep.* **7**, 45559; doi: 10.1038/srep45559 (2017).

**Publisher's note:** Springer Nature remains neutral with regard to jurisdictional claims in published maps and institutional affiliations.

## Supplementary Material

Supplementary Information

## Figures and Tables

**Table 1:** Clinical characteristics of the volunteers.

	Gender % (Prevalence)	Age (years) (mean ± SD)	BMI kg/cm^2^ (mean ± SD)	Secretor positive % (Prevalence)
Male	37.15% (13/35)	40.43 ± 7.34	26.25 ± 3.50	61.54% (8/13)
Female	62.85% (22/35)	37.10 ± 9.95	21.77 ± 2.52	86.36% (19/22)
Total	100% (35/35)	38.43 ± 9.14	23.59 ± 3.68	77.14% (27/35)
