# Supplementary material for: Relevance of secretor status genotype and microbiota composition in susceptibility to rotavirus and norovirus infections in humans
Source: Sci Rep. 2017 Mar 30;7:45559. doi: 10.1038/srep45559 (PMC5372083; doi:10.1038/srep45559)
Supplement: Supplementary Information [file srep45559-s1.pdf]

Relevance of secretor status genotype and microbiota composition in susceptibility to rotavirus and norovirus infections in humans

Jesús Rodríguez-Díaz, Izaskun García-Mantrana, Susana Vila-Vicent, Roberto Gozalbo-Rovira, Javier Buesa, Vicente Monedero and Maria Carmen Collado

Supplementary material

Supplementary material

Figure S1. AnovaPlot with the significant OTU relative abundances between Secretor (n=28) and non-secretor (n=7) individuals. Bars represents the mean of the relative abundances with the standard error. The asterisk indicates the presence of significant differences ($p < 0.05$).

Figure S2. AnovaPlot with the significant OTU relative abundances between FUT2$^{+/+}$ (n=9), FUT2$^{+/-}$ (n=19) and FUT2$^{-/-}$ (n=7). The asterisk indicates the presence of significant differences ($p < 0.05$).

Figure S3. Rarefaction curves based on Chao1 and Shannon indexes in secretor and non-secretor (Panel A and B) and also, in the FUT2 genotypes (Panel C and D). Rarefaction curves are calculated for each sample based on the OTU computations at 97% of homology.

Figure S4. Determination of the differences in the global community (ANOSIM test) (panel A) and bacterial diversity and richness between women and men participating in the study (panel B).

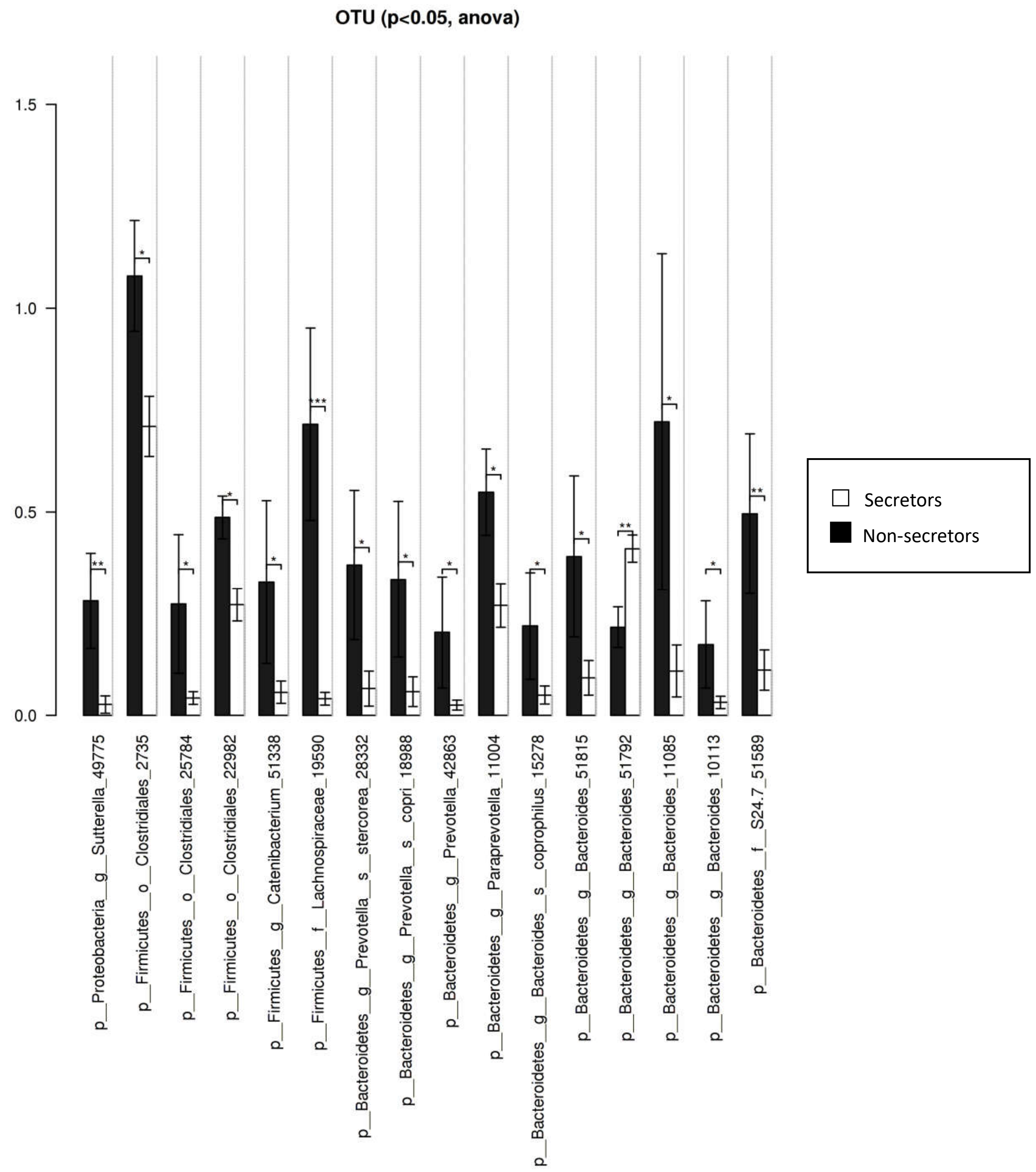

Taxa	P
p__Firmicutes__f__Lachnospiraceae_19590	0.000007
p__Proteobacteria__g__Sutterella_49775	0.001400
p__Bacteroidetes__g__Bacteroides_51792	0.007100
p__Bacteroidetes__f__S24.7_51589	0.008700
p__Firmicutes__o__Clostridiales_22982	0.011000
p__Bacteroidetes__g__Bacteroides_11085	0.018000
p__Firmicutes__o__Clostridiales_25784	0.019000
p__Bacteroidetes__g__Prevotella__s__stercorea_28332	0.021000
p__Bacteroidetes__g__Paraprevotella_11004	0.021000
p__Firmicutes__o__Clostridiales_2735	0.023000
p__Bacteroidetes__g__Prevotella_42863	0.024000
p__Firmicutes__g__Catenibacterium_51338	0.026000
p__Bacteroidetes__g__Prevotella__s__copri_18988	0.029000
p__Bacteroidetes__g__Bacteroides_51815	0.029000
p__Bacteroidetes__g__Bacteroides_10113	0.031000
p__Bacteroidetes__g__Bacteroides__s__coprophilus_15278	0.041000
p__Bacteroidetes__f__S24.7_40924	0.053000
p__Firmicutes__o__Clostridiales_13842	0.054000
p__Firmicutes__f__Christensenellaceae_5674	0.066000

Figure S1

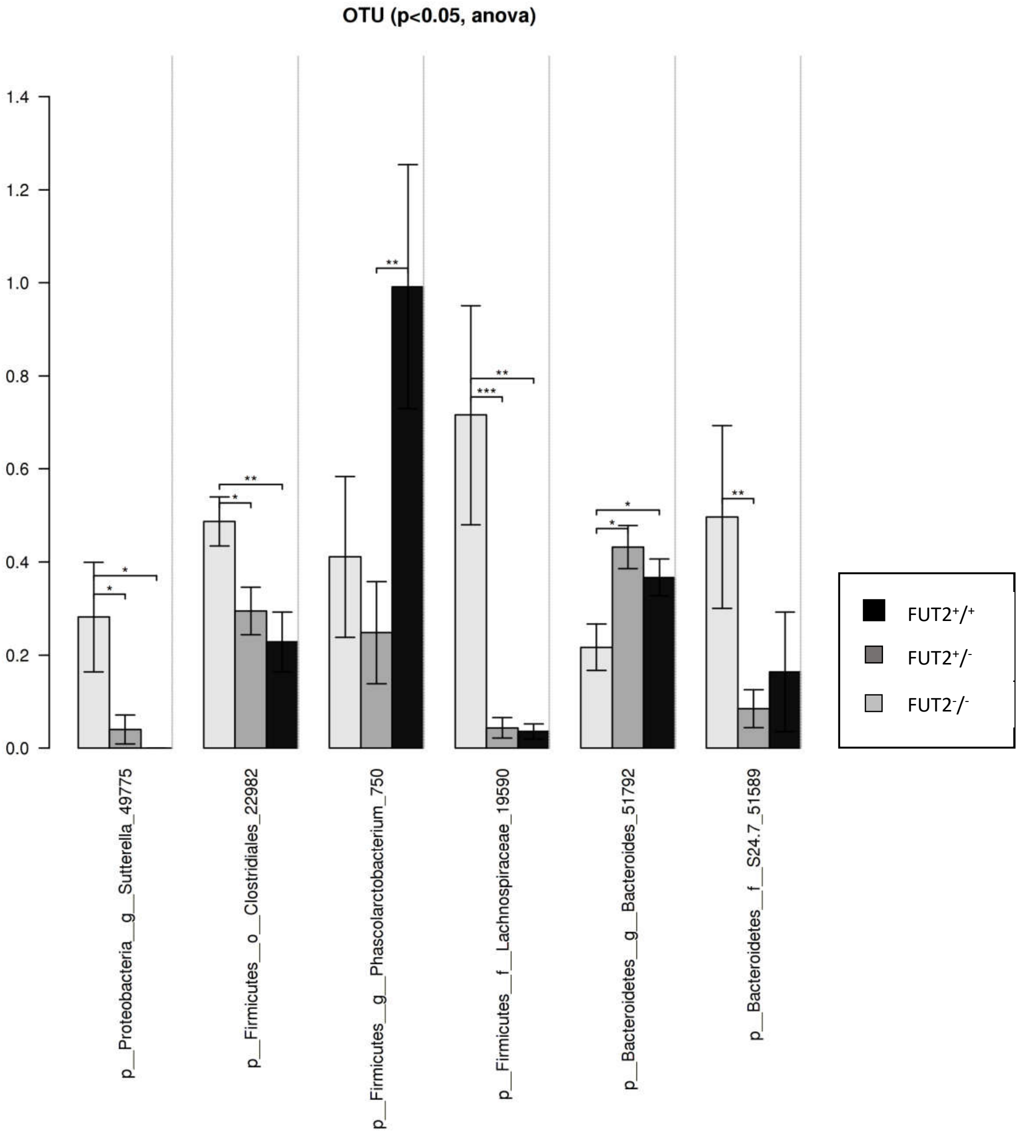

Taxa	P
p__Firmicutes__f__Lachnospiraceae_19590	0.000050
p__Proteobacteria__g__Sutterella_49775	0.005600
p__Firmicutes__g__Phascolarctobacterium_750	0.011000
p__Bacteroidetes__g__Bacteroides_51792	0.018000
p__Firmicutes__o__Clostridiales_22982	0.029000
p__Bacteroidetes__f__S24.7_51589	0.029000

Figure S2

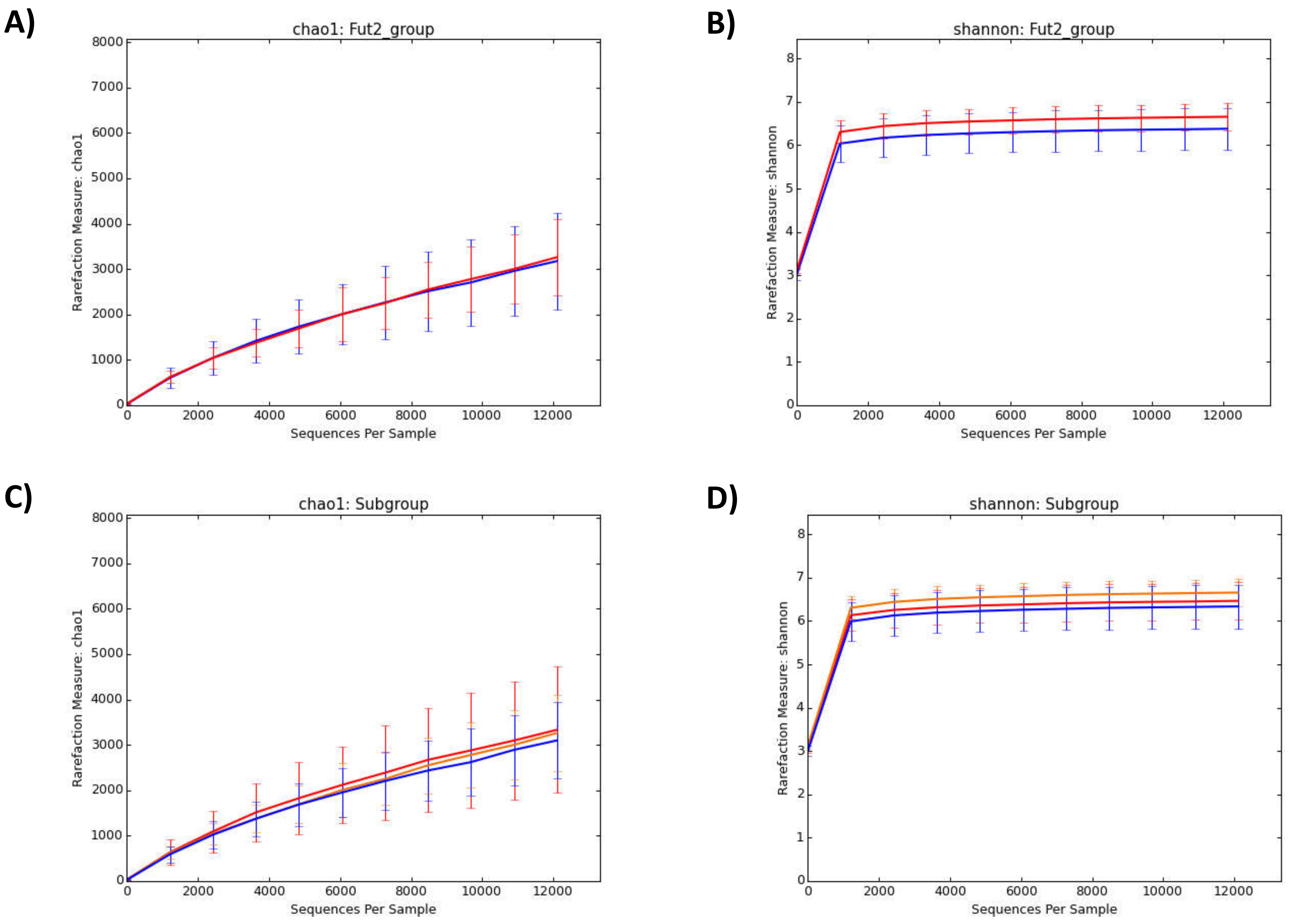
A)
chao1: Fut2_group
Rarefaction Measure: chao1
Sequences Per Sample
B)
shannon: Fut2_group
Rarefaction Measure: shannon
Sequences Per Sample
C)
chao1: Subgroup
Rarefaction Measure: chao1
Sequences Per Sample
D)
shannon: Subgroup
Rarefaction Measure: shannon
Sequences Per Sample

Figure S3

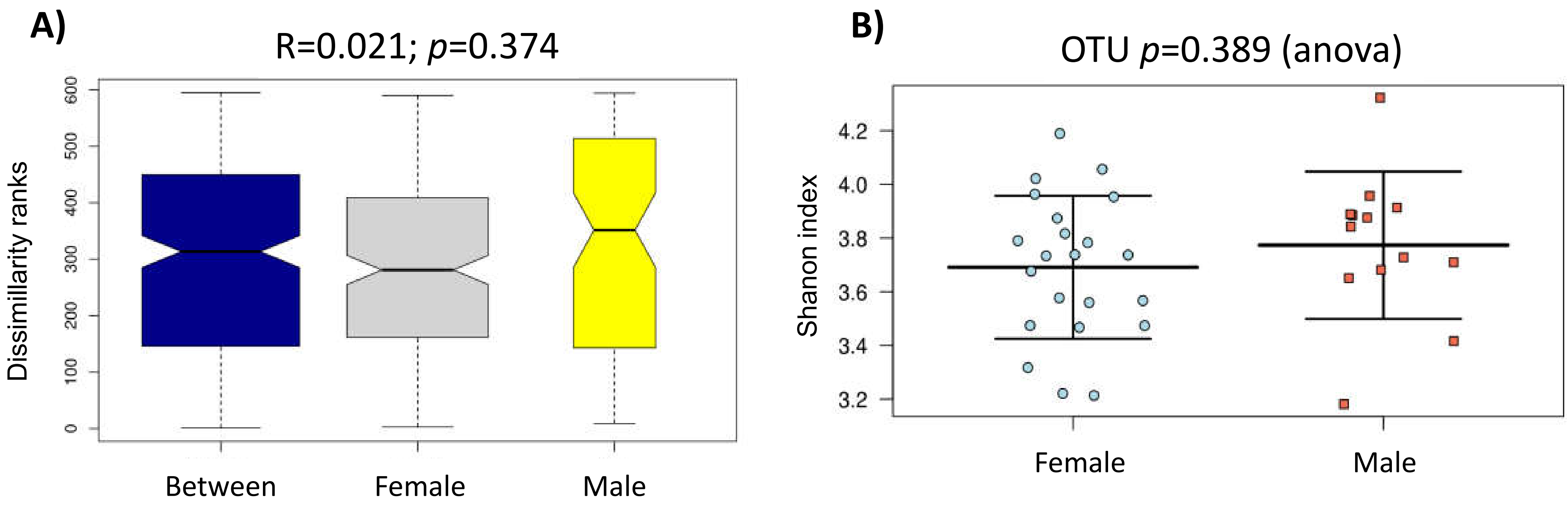
A)
R=0.021; p=0.374
Dissimillarity ranks
0
100
200
300
400
500
600
Between
Female
Male
B)
OTU p=0.389 (anova)
Shanon index
3.2
3.4
3.6
3.8
4.0
4.2
Female
Male

Figure S4
